# Endoscopic Axillary Lymphadenectomy in Breast Cancer: Evolution, Current Evidence, and Future Perspectives

**DOI:** 10.3390/cancers18152520

**Published:** 2026-08-06

**Authors:** Sandra López Gordo, Humberto M. Pontillo Zile, Lidia Blay Aulina, Marta Eguía Larrea, Anna Garcia-Monferrer, Raquel Arranz Jimenez, Isabel Prieto Nieto, Cristina Serra-Serra, Elisa York Pineda

**Affiliations:** 1 General and Digestive Surgery Department, Hospital Universitario de Mataró, 08304 Mataró, Spain; agarciamo@csdm.cat; 2 Unit of Human Anatomy and Embryology, Department of Morphological Sciences, Faculty of Medicine, Universitat Autònoma de Barcelona, 08193 Cerdanyola del Vallès, Spain; 3 General and Digestive Surgery Department, Hospital Comarcal Sant Jaume de Calella, 08370 Calella, Spain; hpontillozile@salutms.cat; 4 General and Gastrointestinal Surgery, Hospital Germans Trias i Pujol, 08916 Badalona, Spain; lblayau.germanstrias@gencat.cat; 5 Department of Surgery, Universitat Autònoma de Barcelona, 08035 Barcelona, Spain; 6 Breast Unit, Department of General and Digestive Surgery, University Hospital of Salamanca, 37007 Salamanca, Spain; meguia@saludcastillayleon.es; 7 Department of Surgery, University of Salamanca, 37008 Salamanca, Spain; 8 HM Santísima Trinidad Hospital, 37007 Salamanca, Spain; 9 Department of General and Digestive Surgery, Hospital Universitario La Paz, 28046 Madrid, Spain; rarranzj@salud.madrid.org (R.A.J.); iprieto@intermic.com (I.P.N.); elisa.york@salud.madrid.org (E.Y.P.); 10 Ginecology Department, Hospital Universitario de Mataró, 08304 Mataró, Spain; cserra@csdm.cat; 11 Breast Pathology Center, Fundación Tejerina, 28003 Madrid, Spain

**Keywords:** breast cancer, axillary lymph node dissection, endoscopic axillary lymphadenectomy, minimally invasive axillary surgery, robotic axillary surgery, fluorescence-guided surgery

## Abstract

Axillary lymph node staging remains a cornerstone of the surgical management of breast cancer. Conventional axillary lymph node dissection (ALND) has historically been the standard procedure for evaluating nodal involvement; however, it is associated with significant morbidity, including lymphedema, sensory disturbances, and reduced shoulder mobility. Over the past few decades, minimally invasive surgery has demonstrated clear benefits in multiple surgical fields, particularly in reducing postoperative morbidity, improving recovery, and enhancing cosmetic outcomes. In breast surgery, these principles have been progressively incorporated into clinical practice. Endoscopic techniques were first introduced in the late 1990s as a minimally invasive alternative to axillary lymph node dissection. Early studies have demonstrated the technical feasibility of endoscopic axillary lymphadenectomy (EALND), reporting adequate lymph node retrieval and acceptable perioperative outcomes. Although the widespread adoption of sentinel lymph node biopsy and the progressive de-escalation of axillary surgery have limited the diffusion of this approach, the potential advantages of minimally invasive techniques appear to be applicable to axillary surgery. In recent years, minimally invasive approaches, including endoscopic and robot-assisted procedures, have been expanded in breast surgery and are now being explored for axillary management. Advances in instrumentation, optics, and imaging technologies, such as fluorescence, have improved surgical visualization and may facilitate safer and more precise dissection of axillary structures. This review summarizes the historical development, technical evolution, and current evidence regarding endoscopic axillary lymphadenectomy for breast cancer. Furthermore, we discuss the potential role of this approach in the modern era of axillary de-escalation and highlight the emerging technologies that may contribute to the future development of minimally invasive axillary surgery.

## Simple Summary

Axillary lymphadenectomy for breast cancer remains a necessary surgical procedure in selected cases. Associated morbidity includes lymphedema, pain, and sensory or mobility impairments. Within the realm of breast cancer innovation, minimally invasive surgery has extended to the axilla through endoscopic axillary lymphadenectomy (EALND). This review aims to summarize the evolution of the technique, clinical outcomes, and current evidence. Available studies suggest that EALND may offer benefits in terms of postoperative morbidity while maintaining adequate surgical outcomes. However, due to the limited number of high-quality studies and the ongoing evolution of this technique, further well-designed research, including studies with longer follow-up, is needed to provide more robust evidence regarding its clinical and oncological outcomes.

## 1. Introduction

Axillary surgery in patients with breast cancer has changed considerably over the past few decades [1]. With the introduction of sentinel lymph node biopsy and a gradual shift towards less aggressive axillary treatments, the number of patients who require axillary lymph node dissection (ALND) has decreased considerably. Simultaneously, improvements in systemic therapies and a deeper understanding of breast cancer biology have paved the way for a more personalized approach to axillary management. However, ALND remains necessary in certain cases, especially for patients who present with a high axillary tumor burden at diagnosis or those who have residual nodal disease after undergoing neoadjuvant treatment (in case of no inclusion in the ALLIANCE A11202 or ADARNAT studies). Axillary staging remains vital for assessing prognosis and shaping adjuvant treatment strategies.

ALND is associated with significant postoperative morbidities. Early complications include seroma formation, sensory disturbances caused by injury or damage to the intercostobrachial nerve, pain, and restriction of shoulder mobility. These sequelae may impair functional recovery and negatively affect the quality of life of patients. In the long term, lymphedema remains the most feared complication, with reported incidence rates varying widely, occurring in approximately 20% of cases [2]. This lymphedema has been described in cases of isolated sentinel lymph node involvement in approximately 5% of cases [2]. The recognition of these complications has been one of the main drivers behind the progressive de-escalation of axillary surgery and the development of less-invasive surgical approaches.

Endoscopic techniques have progressively expanded from breast-conserving surgery and nipple-sparing mastectomy to axillary procedures, reflecting the broader adoption of minimally invasive approaches in breast cancer surgery [3,4].

Initially, axillary lymphadenectomy was performed with liposuction [5] to facilitate the removal of lymph nodes more quickly. However, this made it difficult to identify and count them properly and raised concerns about possible neoplastic dissemination. Therefore, some authors have performed ALND without liposuction [6,7,8,9].

Aponte et al. reported unfavorable results with early endoscopic axillary lymph node dissection and did not recommend its routine use because of prolonged operative times and the lack of a significant reduction in postoperative lymphatic drainage or seroma formation [10]. However, these findings should be interpreted in the context of the technology available at the time. Over the last two decades, advances in surgical instrumentation, particularly the introduction of advanced vessel-sealing devices, have improved the efficiency of axillary dissection by reducing operative time, blood loss, postoperative drainage, and seroma production [11,12]. These technological developments have contributed to improving the outcomes of axillary lymph node dissection and may explain why some of the limitations reported in early endoscopic series are less evident in more recent studies [13].

Expert consensus statements on minimally invasive breast surgery generally recommend endoscopic axillary lymph node dissection (EALND) for patients with clinically node-positive disease (cN1) and no previous axillary surgery [14]. However, the management of the axilla has changed substantially in recent years. In contemporary practice, many patients undergo targeted axillary dissection (TAD) following confirmation of nodal involvement, and the finding of residual nodal disease frequently leads to completion axillary lymph node dissection.

Therefore, restricting the indication for EALND exclusively to patients with cN1 disease may be overly conservative and not necessarily beneficial. Certain patient subgroups, such as those with luminal breast cancer presenting with cN2 disease at diagnosis, may still require axillary lymph node dissection as part of their treatment strategy. From a technical standpoint, there is currently no clear evidence suggesting that a higher nodal burden is a contraindication for an endoscopic approach. Consequently, the indication for EALND should not be based solely on the initial nodal stage but rather on the need for axillary lymph node dissection. The optimal indications and current role of EALND in the era of tailored axillary management will be further explored in the following sections.

The studies conducted to date on EALND are mainly observational, although some comparative studies—including a small number with randomized designs—have also been published [15,16,17,18], demonstrating several of the benefits associated with minimally invasive surgery, including reduced postoperative morbidity and improved functional outcomes. In addition, a meta-analysis including 12 comparative studies and 2157 patients confirmed that endoscopic axillary lymph node dissection was associated with lower intraoperative blood loss, shorter drainage duration, and fewer postoperative complications, while achieving similar lymph node yields compared with conventional axillary lymph node dissection [19].

The main criticism of the endoscopic technique is the increase in surgical time; however, a recent comparative study between the two techniques has shown that endoscopic or robotic axillary lymph node dissection achieves only 9.39 min more on average of surgical time [20].

Notably, a systematic review published in 2009 questioned the oncological safety of endoscopic axillary lymph node dissection because of the limited quality of the evidence available at that time [10]. However, several important studies have been published since then, including comparative studies with long-term follow-up and a recent meta-analysis [19], which considerably strengthened the evidence supporting the safety and effectiveness of this approach. Therefore, we considered it timely to perform an updated review of the literature, bringing together the most relevant studies published to date to provide a comprehensive overview of the evolution of the technique, its clinical outcomes, and its current role in breast cancer surgery.

Taken together, the available evidence supports the technical feasibility of EALND and reports reassuring short-term oncological outcomes; however, its adoption has been constrained by the absence of large prospective trials with mature oncological follow-up. In the current era of axillary de-escalation, its clinical niche has narrowed to patients who still require complete axillary clearance—a subgroup at substantial risk of surgical morbidity—making a minimally invasive alternative potentially relevant. Emerging technologies, such as indocyanine green fluorescence guidance and robotic assistance, may further address some historical limitations of this technique.

The objective of this review is to provide a comprehensive and clinically oriented synthesis of the historical development, technical evolution, perioperative and oncological outcomes, and contemporary role of endoscopic and robotic axillary lymph node dissection in breast cancer.

## 2. Materials and Methods

The present study was designed as a narrative review informed by a structured literature search. Original studies, randomized controlled trials, observational studies, systematic reviews, and meta-analyses published in English were considered. Reference lists of relevant articles were also screened to identify additional publications. Studies were selected according to their relevance to the historical development, surgical technique, perioperative outcomes, oncological safety, and emerging technological innovations in EALND. Studies involving endoscopic axillary surgery performed in combination with breast-conserving surgery or mastectomy were also included in this review. Case reports with insufficient data or non-English manuscripts were excluded.

A literature search was performed using the PubMed, Scopus, and Web of Science databases. Studies published from the earliest available reports of endoscopic axillary surgery (1996) to March 2026 were considered for inclusion. The search strategy included the following terms: ‘endoscopic axillary lymphadenectomy’, ‘minimally invasive axillary surgery’, ‘robotic axillary lymph node dissection’, ‘breast cancer’, and ‘lymph node dissection’.

Given the limited number of available studies and heterogeneity of reported outcomes, a qualitative synthesis of the evidence was performed.

Data extracted included study design, authors, year of publication, sample size, surgical approach, surgical technique and methodology use of liposuction, lymph node yield, operativeoutcomesperioperative morbidity, functional outcomes, recurrence, survival outcomes, and duration of follow-up.

As this study was designed as a narrative review rather than a systematic review, no formal risk-of-bias assessment or PRISMA-based study selection process was performed.

## 3. Historical Evolution of Endoscopic Axillary Surgery

The development of endoscopic axillary surgery has followed a progressive trajectory over nearly three decades, driven by the need to reduce the well-documented morbidity associated with conventional axillary lymph node dissection (CALND) (Table 1). However, it must be noted from the outset that endoscopic axillary lymphadenectomy remains a non-standard technique. The available evidence base consists predominantly of single-center case series, small prospective cohort studies, and a limited number of randomized comparative studies with important methodological limitations, generally with limited follow-up. Furthermore, the progressive de-escalation of axillary surgery, driven by trials such as ACOSOG Z0011 and AMAROS, which have substantially restricted the indications for complete axillary lymph node dissection in early breast cancer, has progressively reduced the patient population to whom complete axillary dissection might be indicated [21,22]. The potential advantages of minimally invasive dissection remain clinically relevant only in this selected subset of patients.

### 3.1. First Descriptions of Axillary Endoscopy and Sampling Techniques (1990s)

The first clinical application of endoscopy in axillary surgery for breast cancer was reported by Salvat et al. in 1996 (Table 2), in what is considered the pioneering publication of this technique [15]. This randomized pilot study compared endoscopic axillary lymph node sampling (axilloscopy) with conventional open surgery in 40 patients with early invasive breast cancer. Using lipoaspiration to create the axillary working space, the authors reported a comparable lymph node yield and equivalent pathological assessment. However, operative time was almost doubled, morphological lymph node damage was more frequent, and two locoregional recurrences occurred in the endoscopic group. The authors concluded that the potential advantages of the technique, particularly regarding postoperative morbidity, remained unproven [15].

At the same time, Suzanne et al. reported a similar lipoaspiration-based approach in a series of 72 patients [23], while Brun et al. subsequently described comparable lymph node retrieval without demonstrating a clear reduction in postoperative complications compared with conventional ALND [5]. Together, these early studies established lipoaspiration as the first-generation technique for creating the endoscopic working space, while highlighting the need for further technical refinement.

The application of endoscopy was later extended to sentinel lymph node biopsy. In 2000, Kühn et al. combined axilloscopy with endoscopic sentinel lymph node detection, reporting an identification rate of 80% using blue dye guidance [34]. This study suggested that endoscopic techniques could be applied not only to complete axillary lymphadenectomy but also to minimally invasive axillary staging.

### 3.2. Development of Endoscopic Lymphadenectomy Techniques

A major concern with first-generation lipoaspiration-based techniques was the theoretical risk that mechanical disruption of the lymph node architecture could mobilize tumor cells. Salvat et al. documented that lipoaspiration produced a significantly higher rate of morphological nodal defects than open surgery [15]. This concern motivated the development of techniques that do not require prior liposuction.

In 1999, Kamprath et al. published the first description of endoscopic axillary lymphadenectomy without prior liposuction, reporting their initial experience in 33 patients [6]. The technique used blunt dissection under CO_2_ insufflation at 10 mmHg pressure. The median operative time was 74.9 min (range 30–130), significantly shorter for the last 17 patients than for the first 16 (*p* < 0.05), confirming a learning curve. No intraoperative complications were recorded, and a median of 14.5 lymph nodes (range, 2–28) were retrieved. Importantly, the median follow-up was only 4.6 months, which the authors acknowledged as insufficient for definitive conclusions regarding oncological safety. Histological examination confirmed that blunt extraction did not produce the nodal architectural damage observed with lipoaspiration [6]. This approach was subsequently replicated by Malur et al. (2001) in 100 consecutive patients, consolidating its technical reproducibility [9].

Tagaya and Kubota (2002) presented a preliminary report on five patients using needlescopic instruments (2 mm diameter) under CO_2_ insufflation, reporting a mean of 13 lymph nodes retrieved, a mean blood loss of 19.4 mL, and no complications over a mean follow-up of 15 months [8]. The small sample size limits the conclusions, but the study demonstrated the feasibility of further miniaturizing the instrument profile.

In 2005, Luo et al. further standardized and refined the EALND technique by describing a reproducible operative protocol that facilitated its dissemination and reproducibility in specialized centers. Subsequent technical refinements have included three-dimensional (3D) endoscopic systems and single-port platforms. The single-port 3D endoscopic technique described by Sae-Lim et al. combines improved depth perception with a reduced incision footprint, although the published experience remains limited to preliminary series with short follow-up [24]. Robotic-assisted approaches are addressed separately in Section 8.

### 3.3. Early Feasibility Studies and Pilot Trials

The evidence base for endoscopic axillary lymphadenectomy primarily rests on feasibility and comparative observational studies. A 20-year systematic review by Mok and Lai (2019) explicitly identified the absence of randomized level I clinical evidence and the lack of long-term follow-up data as the main reasons why endoscopic-assisted breast surgery has not become a mainstream standard of care [3].

A comparative study by de Wilde et al. (2003) allocated 80 patients (40 per arm) to endoscopic or conventional axillary lymphadenectomy using liposuction and CO_2_ insufflation at 8 mmHg with three trocars [17]. A learning curve was documented, with operative times decreasing from a mean of 62 min in the first 50 patients to 36 min. Follow-up was limited to 84 days, precluding the evaluation of oncological outcomes.

The longest prospective follow-up data for endoscopic axillary dissection were provided by Langer et al. (2005), who reported outcomes in 52 patients followed for a median of 71.9 months after endoscopic ALND [25]. This prospective single-center series documented two cases of port-site recurrence (at 24 and 49 months, respectively) and one case of hepatic metastasis, illustrating that adequate long-term follow-up is necessary to capture late oncological events. The lymph node yield was also consistently marginally lower than in the open series, attributed in part to the learning curve and in part to documented node fragmentation during retrieval [15,25].

Hussein et al. (2007) conducted a non-randomized comparison between 16 patients undergoing endoscopic axillary dissection and 25 patients undergoing conventional axillary dissection. The authors reported a mean lymph node yield of 9.3 during the initial 15-case validation phase, which increased to approximately 14 nodes in the subsequent endoscopic series, together with one axillary and breast recurrence after 2 years of follow-up [33]. Short follow-up periods limited oncological conclusions.

Xiong et al. (2020) synthesized data from 12 comparative studies and 2157 patients published between 1996 and 2017 [19]. No statistically significant difference in lymph node yield between mastoscopic and conventional dissection was found (mean difference 0.21; 95% CI −0.62 to 1.05; *p* = 0.62). Mastoscopic dissection was associated with shorter drainage time, shorter hospital stay, and lower complication rates. The methodological heterogeneity of the included studies, variable and often short follow-up periods, and predominance of single-center series are important limitations that restrict the generalizability of these pooled results. Taken together, these studies confirm the technical feasibility of endoscopic axillary dissection and suggest a favorable short-term morbidity profile, while highlighting the need for larger prospective studies with longer oncological follow-up.

### 3.4. Transition from Experimental Techniques to Clinical Application: Emerging Technologies

As the indications for complete axillary lymph node dissection have progressively narrowed, the selected population in whom it remains indicated represents the main target for endoscopic approaches in the future. This population predominantly consists of patients with a high nodal burden at diagnosis or those with residual nodal disease following neoadjuvant chemotherapy who do not achieve a radiological complete response. These patients carry multiple recognized risk factors for lymphedema and upper limb morbidity, including the indication for adjuvant locoregional radiotherapy, among others, and therefore represent the subgroup most likely to benefit from a minimally invasive approach capable of reducing the surgical trauma associated with axillary clearance. Within this group, minimally invasive axillary dissection may offer clinically meaningful advantages in terms of the risk of lymphedema, shoulder function, hospital stay, and cosmesis. The current evidence base consistently supports the technical feasibility and comparable nodal yield [3,6,19,25]; however, endoscopic axillary lymphadenectomy cannot yet be considered standard practice because of the limited number of prospective multicenter studies.

An important emerging development is the incorporation of near-infrared fluorescence imaging with indocyanine green (ICG) into endoscopic axillary procedures, with applications described for sentinel lymph node biopsy and complete lymphadenectomy. Regarding sentinel node identification, two independent retrospective single-center studies evaluated endoscopic ICG-guided sentinel lymph node biopsy without prior liposuction. Wang et al. (2023) compared single-port endoscopic sentinel lymph node biopsy combining ICG and carbon nanoparticles with conventional open biopsy in 60 patients, reporting sentinel node detection rates of 100% in both endoscopic groups versus 95% in the open group, with no significant differences in upper limb function or pain scores [27]. Similarly, Yao et al. (2023) compared single-port non-liposuction near-infrared endoscopic sentinel lymph node biopsy with open surgery in 61 patients, achieving a 100% success rate in both groups; the endoscopic group showed a longer operative time but lower axillary drainage volume, with no differences in bleeding or complications, and no recurrences or metastases during a mean follow-up of 14.6 months [35]. Both studies concluded that endoscopic ICG-guided sentinel node biopsy is not inferior to conventional open biopsy and offers the aesthetic advantage of a single axillary incision through which concurrent breast surgery can also be performed. Regarding complete lymphadenectomy, Larrañaga et al. (2025) described periareolar ICG injection as an integrated step within their standardized 10-step endoscopic axillary lymphadenectomy technique, using fluorescence cameras to visualize lymph node tissue in green during dissection, thereby facilitating the identification of nodal structures and avoiding unnecessary resection of surrounding non-lymphatic tissue [29].

Although prospective evidence specifically evaluating the impact of ICG guidance on oncological or functional outcomes in endoscopic axillary lymphadenectomy is currently lacking, it is reasonable to hypothesize that improved real-time delineation of lymph node boundaries may enhance the selectivity of the dissection, potentially reducing collateral damage to adjacent vascular, nervous, and lymphatic structures, and could therefore contribute to greater oncological completeness and lower procedure-associated morbidity. This remains an area of active development that warrants specific evaluation in future studies.

In summary, the historical arc of endoscopic axillary surgery spans from the first axilloscopic explorations of Salvat et al. (1996) [15] through the elimination of lipoaspiration, standardization of dissection protocols, development of single-port platforms, and fluorescence-guided identification. The available evidence demonstrates consistent technical feasibility and adequate nodal yield but is characterized by limited patient numbers, short follow-up, and predominantly observational design. Its future role will depend on prospective comparative studies with mature oncological endpoints focused specifically on patients who still require complete axillary clearance.

Given the substantial heterogeneity in study design, sample size, surgical technique, and duration of follow-up, the strength of the available evidence varies considerably according to the outcome evaluated. Table 3 provides a critical appraisal of the main evidence domains and highlights the limitations that should be considered when interpreting the reported benefits of EALND.

## 4. Surgical Techniques of Endoscopic Axillary Lymphadenectomy

The first description of EALND appeared in the mid-1990s. The original technique was based on liposuction-assisted dissection; however, this approach was later abandoned. Three axillary low incisions were made, and with the improvement in the technique, CO_2_ was introduced, creating the axillary cavity. Initially, the incisions were 12 mm, which then changed to two incisions of 5 mm and then the optical trocar of 12 mm or 10 mm [5]. The recommended pressure is 8–12 mmHg. Conventional laparoscopic instruments are used during dissection; however, these instruments have been modified and improved over time, incorporating advanced bipolar dissectors that allow for better hemostatic control and sealing. The position of the patient varies depending on the surgeon; initially, the technique was described with the upper limb abducted between 90–130°. With the upper limb abducted and slight flexion of the elbow, the arm is fixed in a support. Using this position and prolonged high abduction, cases of neuropraxia and shoulder pain have been observed; therefore, most authors prefer a position with simple abduction at 90° and a small external rotation of the arm. Some authors place surgical drapes underneath the patient’s scapula to elevate it to 30° [24].

In recent years, since 2023, some authors have described single-port surgical techniques. An incision is made in the anterior or mid-axillary region, approximately at the level of the nipple–areola complex. The incision varied between 2.5 and 3.5 cm in width. [24,26]. The single-port approach facilitates faster visualization of the dorsal neurovascular bundle compared to the three-trocar technique, simplifies the insertion and removal of gauze, and allows for palpation to verify the accuracy of lymphadenectomy. This incision can also be used for endoscopic mastectomy when indicated.

Most authors describe the placement of drains, but a Cochrane review in 2013 shows that their use is not necessary and does not modify the evolution of the seroma, with some authors demonstrating its good evolution [36] in EALND.

Lymphadenectomy should be performed according to established anatomical landmarks. These include the dorsal muscle laterally, thoracic wall medially, neurovascular bundle of the dorsal muscle at its base or floor, axillary vein superiorly, and axillary subcutaneous tissue and pectoralis major muscle superiorly and medially, forming the roof. The periareolar injection of 2.5 mg of indocyanine green and the use of fluorescence imaging cameras are strongly recommended to visualize the lymph nodes in green, thus enabling more precise surgery without excising non-lymphofatty tissue [29]. The green color facilitated visualization, maintaining the lymph node roof-like structure during dissection. Endoscopic dissection provides a better view of the structures than traditional surgery and allows for the identification and preservation of intercostobrachial sensory nerves.

Dissection with advanced bipolar sealants, as previously mentioned, has improved the quality of dissection, reduced blood loss, and resulted in less seroma formation [13].

Extraction of the lymphadenectomy into a bag through three trocars has been described; however, one of the benefits of the single port is skin protection to facilitate the extraction of the specimen without the need to insert a bag.

## 5. Clinical Outcomes

Endoscopic axillary lymph node dissection (EALND), along with its minimally invasive variants, including pure endoscopic, endoscopic-assisted, robotic-assisted, and single-port techniques, has been evaluated against conventional open axillary lymph node dissection (ALND) in comparative studies, meta-analyses, and early clinical series. Current evidence has primarily focused on perioperative outcomes, postoperative morbidity, quality of life, and indirectly, oncological safety within the evolving paradigm of axillary de-escalation.

### 5.1. Feasibility and Operative Time

Since its introduction in the 1990s, EALND has emerged as a technically feasible alternative to conventional ALND. Early reports have demonstrated that the procedure reproduces the fundamental anatomical principles of standard axillary dissection while employing minimally invasive techniques based on tumescent infiltration, liposuction, and video-assisted endoscopic dissection [15,35].

Initially, the widespread adoption of EALND was limited by longer operative times, with procedures typically lasting between 60 and 150 min [15,35]. However, continuous technological advances, including high-definition three-dimensional imaging systems, dedicated endoscopic instrumentation, and increasing surgical experience, have substantially improved procedural efficiency. Contemporary single-port series using three-dimensional visualization report median operative times of approximately 39–46 min, approaching those achieved with conventional open surgery [37,38].

Operative time is influenced by several factors, including body mass index (BMI), axillary nodal burden, surgeon experience, and use of advanced energy devices [4,9,27,37]. Perhaps the greatest technical advantage of EALND is the magnified view of the axillary anatomy, which facilitates a more accurate identification of anatomical structures and enables safer and more meticulous dissection [35].

Surgical proficiency along this learning curve is generally achieved after approximately 20–30 procedures, whereas consistent operative performance requires greater cumulative experience [24,35]. The principal technical challenges relate to the loss of tactile feedback, instrument manipulation within a confined operative field, endoscopic hemostatic control, and three-dimensional anatomical orientation. The introduction of three-dimensional imaging platforms, together with prior experience in laparoscopic surgery, has substantially shortened this learning curve and facilitated the wider adoption of the technique [7].

### 5.2. Lymph Node Retrieval

Available evidence demonstrates that EALND achieves lymph node retrieval comparable to that of conventional ALND. Most published series report the excision of 10–18 lymph nodes, fulfilling the accepted criteria for accurate pathological staging [7,10,19,35].

Comparative studies and multicenter investigations have consistently shown no significant differences between EALND and conventional ALND in terms of lymph node retrieval [7,10,35]. Although early concerns were raised that liposuction might result in lymph node fragmentation, subsequent studies have demonstrated that this does not adversely affect histopathological evaluation or the detection of nodal metastases [10,15].

The incorporation of carbon nanoparticles, indocyanine green (ICG) fluorescence imaging, and advanced image-guidance technologies may further improve lymph node identification during endoscopic dissection, potentially enhancing the accuracy of the procedure [7,19,24].

### 5.3. Conversion to Open Surgery

Current conversion rates are low and generally remain below 5%, particularly in experienced centers performing minimally invasive breast surgery [7,10,24]. The most common reasons for conversion include intraoperative hemorrhage, inadequate visualization of the operative field, severe obesity, extensive axillary fibrosis, bulky or densely adherent nodal disease, and technical difficulties related to surgical instrumentation [10,35].

Although obesity increases technical complexity, some authors have reported satisfactory outcomes in patients with a BMI exceeding 24 kg/m^2^ without major technical limitations [20].

Importantly, no conversions have been reported in the most recent endoscopic series [24,28]. The progressive refinement of surgical techniques, together with increasing surgeon experience, has markedly reduced conversion rates over time. Nevertheless, conversion remains more likely in patients with locally advanced disease or dense axillary fibrosis after neoadjuvant systemic therapy [10,28].

### 5.4. Postoperative Morbidity

Compared with conventional ALND, EALND appears to reduce the incidence of lymphedema, chronic pain, paresthesia, shoulder dysfunction, and sensory disturbances [7,24,35,37].

The lower postoperative morbidity associated with EALND is likely multifactorial and may be attributed to reduced surgical trauma, improved preservation of neural structures, greater precision during axillary dissection, and integration of lymphatic preservation techniques, including axillary reverse mapping [20,24,37].

Seroma remains the most common postoperative complication, with reported rates comparable to or slightly lower than those observed following conventional ALND [10,37]. Intraoperative blood loss is consistently reduced with EALND, largely owing to superior visualization and the routine use of advanced vessel-sealing devices [24,37].

Neurological morbidity also appears to have been reduced. Improved visualization of the axillary anatomy facilitates the identification and preservation of critical neural structures, translating into lower reported rates of paresthesia, chronic pain, and shoulder dysfunction compared to conventional axillary dissection [35,37].

Similarly, published data suggest favorable lymphedema outcomes, with several studies reporting incidences below 5% [4,9]. The addition of axillary reverse mapping (ARM) may further preserve upper limb lymphatic drainage and reduce the risk of postoperative lymphedema [24].

A recent meta-analysis demonstrated lower overall complication rates and reduced intraoperative blood loss following EALND than following conventional open ALND [19].

Comparative studies have also reported lower rates of seroma formation, reduced postoperative drainage, and less hemorrhage following EALND [10,13]. Furthermore, robot-assisted axillary dissection appears to provide additional reductions in blood loss and postoperative lymphatic leakage compared with conventional endoscopic techniques [32].

Major complications, including hematoma, persistent lymphatic leakage, and delayed wound healing, are uncommon and are generally managed successfully with conservative treatment.Short term endoscopic series have also reported a low incidence of postoperative lymphedema [7,20,24,39].

### 5.5. Postoperative Recovery

Compared with conventional ALND, EALND is associated with less surgical trauma, reduced postoperative pain, and faster functional recovery times. Several studies have demonstrated earlier restoration of shoulder mobility, less functional impairment, and a more rapid return to normal daily activities following the endoscopic approach [35,37].

The duration of postoperative drainage and length of hospital stay are generally comparable to or slightly shorter than those observed after conventional surgery, although these outcomes remain largely dependent on institutional postoperative protocols [37].

Interestingly, several specialized centers have reported performing EALND without routine drain placement, achieving favorable postoperative outcomes without increasing complication rates compared with conventional drainage strategies [36,40].

Overall, EALND is associated with lower drainage volumes, shorter drainage duration, and reduced hospital stay compared with conventional ALND. These findings are consistent with the recognized benefits of advanced vessel-sealing technologies in open axillary surgery [7,19].

### 5.6. Cosmetic Outcomes

Superior cosmetic outcomes represent one of the most consistent advantages of EALND, driven by smaller, well-hidden incisions that reduce scar fibrosis and improve the patient-reported quality of life [15,24,37]. Comparative studies have confirmed better cosmetic results, improved breast symmetry, and greater patient satisfaction than conventional ALND [7,16,24], an advantage that is particularly pronounced among younger women and those undergoing breast-conserving surgery or immediate reconstruction [1,4,9]. Single-port techniques may further enhance this benefit by allowing both breast and axillary procedures to be performed through a single, discreetly positioned incision [24].

## 6. Oncological Safety

Available evidence reports reassuring oncological outcomes following EALND, including low rates of axillary recurrence in published series. However, direct comparison with conventional ALND remains limited by the small number of randomized studies, selected patient populations, and heterogeneous follow-up. Studies with longer follow-up have reported axillary recurrence rates of approximately 1–3% [10].

Nevertheless, the current evidence remains limited by several factors, including the limited number of randomized controlled trials, short or intermediate follow-up, and the predominance of patients with a low axillary tumor burden [10].

Consequently, robust contemporary evidence confirming the oncological safety of EALND in patients with advanced axillary disease remains lacking.

Available series with short- and mid-term follow-ups have not demonstrated increased rates of locoregional recurrence or distant metastasis following EALND [7,24]. Available oncological outcomes are reassuring; however, the current level of evidence remains low because of selected patient populations, heterogeneous follow-up, and the scarcity of large prospective studies with mature long-term oncological outcomes. Therefore, definitive oncological equivalence between EALND and conventional ALND cannot currently be established.

## 7. Robotic-Assisted Axillary Lymph Node Dissection

The use of robotic technology has expanded across multiple surgical specialties, offering potential advantages such as instrument stability, articulated motion, tremor filtration, high-definition three-dimensional vision, and improved surgeon ergonomics [41].

In breast surgery, procedures such as robotic mastectomy have been standardized in recent years [42,43]; however, evidence regarding robotic-assisted axillary lymph node dissection (RALND) remains limited. Ahn et al. compared robotic and conventional axillary lymph node dissection in 32 patients overall undergoing robot-assisted nipple-sparing mastectomy and found no significant differences in the number of retrieved lymph nodes, perioperative outcomes, or postoperative complications, including lymphedema and shoulder dysfunction [30]. The robotic approach was associated with smaller incisions and comparable surgical safety. Although experience with robotic axillary dissection is still scarce, these findings suggest that robot-assisted approaches may achieve oncological and surgical outcomes similar to those reported for both endoscopic and conventional open axillary dissection.

Additional evidence was provided by Chen et al., who compared da Vinci robot-assisted axillary lymph node dissection with conventional ALND in 60 breast cancer patients. The robotic approach was associated with lower rates of wound infection, fat necrosis, and upper limb lymphedema while maintaining adequate lymph node retrieval and excellent cosmetic results. However, it was limited by a relatively short follow-up period and small sample size [31]. Other studies have shown similar results, with a better functional impact on the upper limbs [32].

The main limitation of robotic surgery remains its high initial cost, owing to the purchase price of the platform, annual maintenance, and use of specialized instruments. However, these costs could be partially offset by a reduction in complications, decreased need for secondary procedures, improved quality of life, and shared use of the platform among different surgical specialties [44].

Whether the additional costs of robotic surgery are justified by improvements in patient outcomes remains unclear.

As previously explained, robotic ALND requires access to an expensive surgical platform, specialized instruments, and trained, qualified personnel who have performed a minimum number of robotic surgeries. Operative time increases during the initial learning phase, and studies have yet to demonstrate a significant benefit over endoscopic ALND.

Furthermore, there are no robust studies regarding the cost-effectiveness of RALND. For these reasons, robotic ALND should be considered an emerging approach in specialized, high-volume centers rather than a standard alternative to conventional ALND.

## 8. Current Role in the Era of De-Escalation of Axillary Surgery

Building on the de-escalation rationale outlined in the Introduction, the role of EALND in routine clinical practice has been further reshaped by a series of trials that directly address the extent of axillary surgery required [22,40].

Landmark clinical trials, including ACOSOG Z0011, AMAROS, IBCSG 23-01, SENOMAC, SOUND, and INSEMA, have demonstrated that many patients with limited axillary nodal involvement can safely avoid ALND without compromising locoregional control, disease-free survival, or overall survival [29,45].

Furthermore, the increasing use of axillary radiotherapy and targeted axillary dissection following neoadjuvant systemic therapy has further reduced the need for completion of ALND.

Nevertheless, a subset of patients continues to require complete axillary dissection for treatment. Considering the potential benefits of minimally invasive axillary surgery and the encouraging, although still limited, evidence regarding its oncological outcomes, EALND may represent an attractive alternative in the following scenarios: young patients with high cosmetic expectations, patients undergoing endoscopic or robotic breast surgery, patients in whom completion ALND remains indicated according to current treatment guidelines, patients with residual nodal disease following neoadjuvant systemic therapy, and high-volume breast units with expertise in minimally invasive and robotic breast surgery [24,29,40].

The future integration of robotic platforms, fluorescence-guided surgery [46], artificial intelligence-assisted image analysis, and image-guided axillary procedures may further refine the precision and clinical applicability of EALND.

Therefore, EALND should not be considered an alternative to current axillary de-escalation strategies, but rather an alternative surgical approach when complete ALND remains indicated. Its potential target population includes selected patients with persistent clinically significant nodal disease, patients with residual nodal disease after neoadjuvant systemic therapy when completion ALND remains indicated, and patients with a higher axillary tumor burden who do not meet criteria for omission of ALND. In these patients, a minimally invasive approach may be particularly relevant to reduce postoperative morbidity associated with complete axillary dissection. The exact proportion of breast cancer patients who may currently be eligible for EALND is difficult to estimate, as indications for ALND continue to evolve according to tumor burden, response to neoadjuvant treatment, radiotherapy strategies, and ongoing de-escalation trials. Nevertheless, these patients are expected to represent an increasingly selected subgroup of the breast cancer population.

In the era of de-escalation, the performance of ALND in the clinical setting will not depend on increasing the number of possible ALND procedures but rather on improving outcomes when it remains necessary. Although the available evidence supports the technical feasibility of EALND and suggests potential benefits in postoperative morbidity, its limited methodological quality and the lack of mature long-term oncological data have restricted its widespread adoption into routine clinical practice. Consequently, EALND is currently more appropriately considered a potential alternative in selected patients and centers with experience and technical skills in endoscopic surgery. Nevertheless, the increasing incorporation of endoscopic and robotic techniques into breast surgery may progressively expand the number of surgical teams with the skills required to implement EALND.

The conventional ALND technique with the largest body of published literature—albeit with the limited evidence previously discussed—is the three-port lymphadenectomy. However, other surgical techniques—such as single-port surgery, the use of indocyanine green, or even robotic approaches—are supported by less scientific evidence due to the small number of studies, which consist primarily of case series.

## 9. Limitations and Critical Appraisal of Current Evidence

This review has several limitations. The included studies span from the 1990s to the present and use heterogeneous methodologies and surgical techniques. Many studies include small patient populations and relatively short follow-up periods.

Despite three decades of evidence regarding EALND, the quality of the scientific evidence remains limited. Most studies are retrospective or prospective single-center series, typically involving a small number of patients. Few studies are truly randomized, and those were conducted several years ago—prior to the introduction of contemporary technological advances that facilitates endoscopic dissection.

Consequently, the available literature is particularly useful for establishing the technical feasibility of EALND, but provides a lower level of certainty regarding its potential superiority in postoperative morbidity and, especially, its long-term oncological safety.

A clear limitation is the heterogeneity of the studies regarding technique—such as the method of creating the surgical space, liposuction, CO_2_ insufflation, trocars used, and even the type of energy or robotic platforms.

Furthermore, perioperative outcomes such as seroma, lymphedema, pain, shoulder dysfunction, and functional recovery have not been uniformly defined or assessed. These differences limit direct comparisons between studies and may partly explain discrepancies in reported outcomes.

Importantly, the available studies should also be interpreted within their technological context. Early studies published during the initial development of EALND largely reflect the feasibility and learning curve of the technique using the endoscopic equipment available at that time. In contrast, contemporary studies incorporating high-definition imaging, advanced energy devices, fluorescence guidance, single-port platforms, and robotic assistance should not be directly compared with these early experiences, although their evidence remains limited by predominantly small and observational study designs.

The learning curve represents another important source of potential bias. Several early studies documented progressive reductions in operative time and improvements in lymph node retrieval as surgical experience increased. Conversely, most contemporary studies originate from specialized centers with expertise in minimally invasive breast surgery. Favorable results obtained in these settings may therefore not be directly reproducible in lower-volume centers, limiting the external validity of the available evidence.

The evidence regarding postoperative morbidity is encouraging but should also be interpreted cautiously. Comparative studies and meta-analyses suggest potential reductions in blood loss, drainage duration, postoperative pain, lymphedema, and impairment of shoulder function. However, much of this evidence derives from observational studies with heterogeneous outcome definitions and variable follow-up. Therefore, the available data suggest a potential morbidity benefit rather than establishing the superiority of EALND over conventional ALND.

The greatest uncertainty concerns long-term oncological safety. Although the available studies report reassuring rates of axillary recurrence and no clear evidence of inferior oncological outcomes compared with conventional ALND, the number of randomized studies is small, patient populations are highly selected, and mature long-term oncological follow-up is lacking in much of the literature. This limitation is particularly relevant for patients with a high nodal burden or residual nodal disease following neoadjuvant systemic therapy, who represent an important proportion of the contemporary population still requiring complete ALND. Consequently, current evidence is insufficient to establish definitive oncological equivalence between EALND and conventional ALND.

Publication bias should also be considered. Much of the literature originates from centers actively developing or routinely performing minimally invasive axillary surgery, and favorable experiences may be more likely to be reported than unsuccessful implementation, conversions, or negative outcomes.

Finally, interpretation of historical EALND studies within contemporary breast cancer practice is challenging. Progressive axillary de-escalation means that many patients included in early EALND studies would no longer undergo complete ALND today. Therefore, future research should specifically evaluate the smaller contemporary population in whom complete axillary clearance remains indicated, ideally through prospective multicenter studies using standardized definitions of morbidity, patient-reported outcomes, cost-effectiveness analyses, and sufficiently long oncological follow-up.

Table 3 summarizes the strength of the current evidence across the principal clinical domains and highlights the main limitations affecting its interpretation.

## 10. Future Perspectives

As previously mentioned, the incidence of ALND has been declining in recent decades, and available evidence suggests that the minimally invasive approach may offer benefits in reducing postoperative morbidity compared with conventional ALND [16].

Simultaneously, the increasing adoption of de-escalation strategies, including targeted axillary dissection and, in selected patients, omission of sentinel lymph node biopsy, continues to redefine the role of axillary surgery.

Technological advances are expected to further improve the feasibility and reproducibility of this procedure. Recent reports have demonstrated the successful application of single-port and three-dimensional endoscopic systems, providing improved visualization and reducing the number of surgical incisions [27]. Similarly, robot-assisted axillary surgery offers potential advantages, including enhanced dexterity, articulated instruments, tremor filtration, and high-definition three-dimensional vision, which may facilitate the dissection of complex axillary anatomy [30].

The use of fluorescence-guided techniques has facilitated the visualization of lymph nodes during the procedure; however, there remains a vast field of research in this area. Pre- or post-surgical lymphographic mapping, reverse mapping, and new molecules can help guide us visually and in a more targeted manner.

Future research should focus on defining the optimal indications for EALND, evaluating its integration with image-guided technologies and robotic platforms, and assessing its impact on functional outcomes, quality of life and long-term oncological safety.

**Figure 1:**
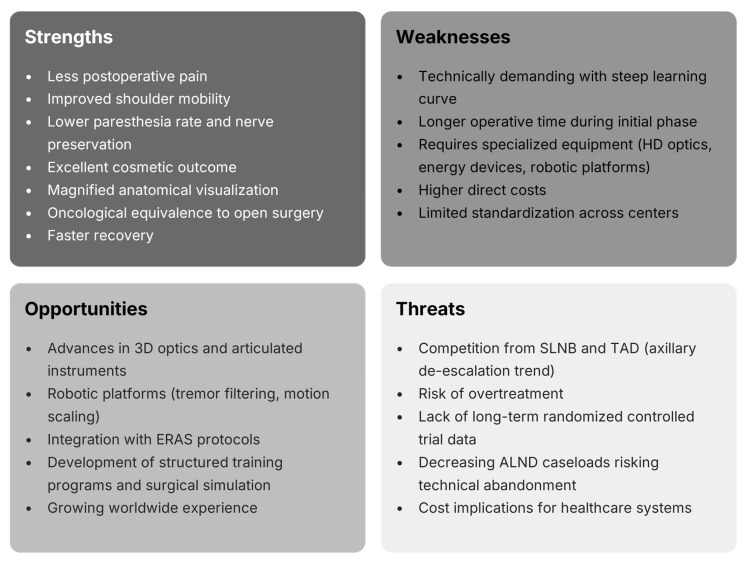
Summary of the current strengths, weaknesses, opportunities, and threats of endoscopic axillary lymph node dissection (EALND).

Figure 1 summarizes the current evidence and future directions, along with the main strengths, weaknesses, opportunities, and threats associated with EALD.

## 11. Conclusions

The progressive de-escalation of axillary treatment has substantially reduced the number of patients requiring an axillary lymph node dissection. Nevertheless, whenever ALND remains indicated, minimally invasive approaches have demonstrated several advantages over conventional surgery, particularly in reducing postoperative morbidity while maintaining oncological safety.

This review suggests that EALND is a technically feasible minimally invasive alternative for selected patients requiring complete ALND and may provide benefits in postoperative morbidity, upper limb function, quality of life, and cosmetic outcomes. However, these findings should be interpreted cautiously, as the available evidence is predominantly based on small observational studies, heterogeneous surgical techniques, and limited long-term oncological follow-up. Therefore, although the available data are encouraging, further high-quality prospective studies are required before definitive conclusions regarding its superiority in postoperative morbidity or long-term oncological safety can be established. These potential benefits may be particularly relevant for the selected subgroup of patients who continue to require complete axillary clearance in the current era of axillary de-escalation. Beyond demonstrating technical feasibility, endoscopic axillary lymphadenectomy has laid the foundation for subsequent developments in robotic surgery, fluorescence-guided lymphatic mapping, and image-guided procedures. Future prospective studies with long-term oncological follow-up are needed to better define the optimal indications and clinical role of minimally invasive axillary surgery in the modern era of personalized and de-escalated axillary management.

## Figures and Tables

**Table 1:** Technical evolution of endoscopic axillary lymph node dissection (EALND) across four chronological eras, from liposuction-assisted dissection (1996–1998) to single-port three-dimensional and robotic platforms (2020–present). Key technical milestones and primary references are indicated for each period.

**1996–1998 | Liposuction-Assisted EALND**	
• Patient supine with slight lateral displacement; operated arm horizontal at 90°, remaining mobile throughout procedure • Axillary infiltration with tumescent lipolysis solution (saline + distilled water + xylocaine/epinephrine); 20 min wait; lipoaspiration with Karman 8 mm cannula at 0.8–1 bar for 10 min; aspirated fluid sent to pathology • 10 mm optical trocar at anterior axillary line (equatorial breast level), secured with purse-string suture; CO_2_ at 8 mmHg; two accessory 5 mm ports at lateral border of pectoralis major and anterior border of latissimus dorsi • Dissection with monopolar scissors (right hand) + atraumatic forceps (left hand); small vessel coagulation; lymph node extraction with Dargent coelioextractor or endobag • Final palpatory check through optical port incision—no residual lymph node should be palpable; routine suction drain placement	References: - Salvat, J. et al. [15] Eur. J. Obstet. Gynecol. Reprod. Biol. 1996, 70(2), 165–173. - Suzanne, F. et al. [23] Chirurgie 1997, 122(2), 138–142. - Brun, J.L. et al. [5] Eur. J. Surg. Oncol. 1998, 24(1), 17–20.
**1999–2005 | CO_2_-Assisted EALND: From Liposuction to Gasless Techniques**	
• Three coexisting approaches for working space creation: (1) CO_2_ with blunt dissection without liposuction; (2) CO_2_ with prior liposuction; (3) external mechanical retraction without CO_2_ • Without liposuction (Kamprath 1999): 10 mm trocar caudal in anterior axillary line; CO_2_ at 10 mmHg; blunt dissection with 10 mm optic; special 8.5 cm trocars (Carl Storz); trocar fixation with catgut 0; Robinson 18-Ch drain through caudal trocar • Needlescopic variant (Tagaya 2002): preperitoneal distension balloon for initial space; CO_2_ at 6–8 mmHg; 2 mm instruments; all neurovascular structures preserved without injury • External retractor without CO_2_ (Lim & Lam 2002): mechanical retraction as alternative to insufflation; periareolar and lateral chest incisions; 2D 30° camera • With liposuction (Luo 2005): axilla appears as “cobweb-like” reticular structure after insufflation, with lymph nodes hanging from fibrous strands; dissection with diathermy scissors following the cords; 10 mm midaxillary incision reused as optical trocar port; CO_2_ at 8 mmHg	References: - Kamprath , S. et al [6]. Surg. Endosc. 1999, 13(12), 1226–1229. - Malur, S. et al [9]. Surg. Laparosc. Endosc. Percutan. Tech. 2001, 11(1), 38–41. - Tagaya, N., Kubota, K [8]. Surg. Endosc. 2002, 16(2), 307–309. - Lim , S.M., Lam, F.L. Ann. J [24]. Surg. 2005, 190(4), 641–643. - de Wilde, R.L. et al [17]. J. Am. Assoc. Gynecol. Laparosc. 2003, 10(1), 75–79. - Luo, C. et al. Surg. Laparosc. Endosc. Percutan. Tech. 2005, 15(3), 153–159.
**2005–2020 | Standardized Mastoscopic EALND (MALND)**	
• Formal standardised protocol: patient supine, arm abducted 90° (forearm hanging along bed frame); CO_2_ at 8 mmHg; 30° 10 mm endoscope; 3-port axillary setup; arm abduction > 90° associated with neuropraxia and avoided in later series • Systematic identification and preservation of four critical structures: intercostobrachial nerve, lateral thoracic artery, medial thoracic nerve, thoracoepigastric vein; endoscopic magnification (×7) facilitates nerve identification more precisely than open surgery • Dissection from centre to axillary apex until axillary vein identified; then bilaterally downward; axillary lavage with warm distilled water at end of procedure • Introduction of advanced bipolar vessel-sealing devices (LigaSure, ultrasonic systems): demonstrated reduction in blood loss, drainage volume and seroma formation • Large-scale comparative evidence: 996 patients across 15 institutions; meta-analysis of 12 studies (2157 patients) confirming equivalent nodal yield and lower morbidity vs. conventional ALND	References: - Luo, C. et al. Surg. Laparosc. Endosc. Percutan. Tech. 2005, 15(3), 153–159. - Luo, C. et al [16]. Mayo Clin. Proc. 2012, 87(12), 1153–1161. - Xiong, H. et al [19]. Cancer Control. 2020, 27, 1073274820932987. - Langer, I. et al [25]. Breast Cancer Res. Treat. 2005, 90(1), 85–91. - Cordatellas, T. et al [13]. Int. J. Surg. 2011, 9(8), 636–640. - Wienerroither, V. et al [11]. BMC Surg. 2022, 22(1), 7. - Lumachi, F. et al [12]. Breast Cancer Res. Treat. 2013, 142(2), 399–404.
**2020–Present | Single-Port 3D EALND + ICG Fluorescence/Robotic EALND**	
• Single axillary incision 2–3 cm; glove-made single port (Alexis wound protector + surgical glove + 1 × 12 mm + 2 × 5 mm trocars) or commercial Glove Port (Nelis); 3D 30° endoscope (Karl Storz TIPCAM 1S 3D VIDEO); CO_2_ at 12 mmHg • Axillary cavity conceptually divided into 4 quadrants by intersection of lateral thoracic vein and second intercostobrachial nerve; specimens extracted directly through single incision without endobag; skin protected by wound protector • Periareolar ICG injection 0.3 mg/mL + near-infrared fluorescence at 760 nm (FloNavi™): real-time green visualisation of lymph nodes; improves selectivity and precision of dissection; endoscopic ICG-guided sentinel node biopsy: 100% detection vs. 95% open surgery • Robotic EALND (da Vinci Xi): 3D HD vision, articulated wristed instruments, tremor filtration; two robotic arms via auxiliary trocars (bipolar + hook/ultrasonic); CO_2_ 8–12 mmHg; trocar fixation with silk suture; Firefly ICG fluorescence integrated in robotic camera	References: - Sae-Lim, C., Lai, H.W. et al [24]. Breast J. 2024, 2024, 6319218. - Roufael, J. et al [26]. Gynecol. Obstet. Fertil. Senol. 2025. - Wang, Z.H. et al [27]. Surg. Endosc. 2023, 37(10), 7591–7599. - Yao, C. et al [28]. World J. Surg. Oncol. 2023, 21, 66. - Larranaga, J. et al [29]. Cir. Esp. (Engl. Ed.). 2025, 103(5), 308–309. - Ahn, J.H. et al [30]. Breast Cancer Res. Treat. 2023, 198(3), 405–412. - Chen, K. et al [31]. Int. J. Med. Robot Comput. Assist. Surg. 2021, 17(6), 1–9. - Wu, Z. et al [32]. World J. Surg. Oncol. 2025, 23(1).

**Table 2:** Published studies on endoscopic axillary lymph node dissection (EALND): case series and comparative studies. Data correspond to the EALND/minimally invasive arm only for comparative studies. NR: not reported.

Author (Year)	Country	Study Design	n (EALND)	Liposuction	CO_2_ (mmHg)	Op. Time (min)	LN Harvested	Blood Loss (mL)	Seroma	Lymphedema	Sensory Loss	Shoulder Restriction	Conversion to Open	Recurrence/ Oncological	Follow-Up
Salvat et al. [15]	France	RCT pilot	20	Yes	8	60.9	12.9	NR	25% (5/20)	NR	NR	NR	NR	2 locoregional relapses	NR
Suzanne et al. [23]	France	Prospective series	72	Yes	NR	50	14.5 (7.5 asp + 7.0 inst.)	NR	2.8%	NR	2.8% (transient)	8% at 6 m; 0% at 18 m	NR	NR	NR
Brun et al. [5]	France	Prospective series	43	Yes	4–8	85–100	13.6 (2.5 asp + 11.1 inst.)	NR	9%	0%	NR	NR	NR	NR	18 months
Kamprath et al. [6]	Germany	Prospective series	33	No	10	74.9 (30–130)	14.5 (2–28)	18.6 cc	10% (3/33)	NR	27.2% (C5 derm.)	0% at 4.6 m	NR	NR	4.6 months
Malur et al. [9]	Germany	Prospective series	100	No	10	75	16	20	4%	1%	14%	2%	NR	NR	NR
Tagaya & Kubota [8]	Japan	Pilot series	5	No	6–8	105.4	13	19.4	0%	0%	0%	0%	0%	NR	15 months
Hüscher et al. [18]	Italy	RCT pilot	10	No	NR	168 (130–210)	23 (14–41)	NR	22% (2/10)	NR	0%	NR	0%	0%	43–70 months
de Wilde et al. [17]	Germany	Comparative (EALND vs. CALND)	40	Yes	8	62 (post-LC: 36)	11 (4–21)	NR	90%	NR	NR	Lower vs. open at 3 months	NR	NR	84 days
Langer et al. [25]	Switzerland	Prospective single-center series	52	NR	NR	NR	NR	NR	NR	NR	NR	NR	NR	2 port-site recurrences; 1 hepatic metastasis	Median 71.9 months
Hussein et al. [33]	Egypt	Comparative (EALND vs. CALND)	16	No	Yes	Phase I: NR; Phase II: NR	Phase I: 9; Phase II: 14	NR	NR	NR	NR	NR	2 (13%)	1 axillary + breast recurrence	32 months
Luo et al. (2008)	China	Prospective series	522	Yes	8	39.2 (20–156)	13.4 (4–38)	Minimal (<10)	0%	NR	NR	NR	0%	0% axillary relapse/trocar implantation	26.3 months
Luo et al. [16]	China	Phase III multicenter RCT (MALND vs. CALND)	496	Yes	8	40.63 ± 14.27	17.65 ± 5.38	12.82 ± 5.46	4.8% (24/496)	Lower vs. CALND	Lower vs. CALND	Similar	27 (5.4%)	Axillary recurrence 1.2%; DFS 64.5%; OS 81.7%	Median 63 months
Chen et al. (2020)	China	Retrospective series	16	NR	Yes	ALND: 51.1	15.75	29.3	0%	1/16 (mild, resolved)	1/16 (resolved at 3 m)	0%	NR	0% at 28 m	28 months
Ahn et al. [30]	Korea	Comparative (RALND vs. CALND)	11	No	Yes	47 (25–86)	15 (3–22)	70	NR	9.1%	NR	27.3%	1 (vein tear)	NR	19 months (lymphedema)
Chen K et al. [31]	China	Comparative (dvALND vs. CALND)	30	No	8–12	50.4	NR	10.3	NR	6.7%	NR	NR	NR	0% at 3 m	3 months
Sae-Lim & Lai et al. [24]	Thailand/Taiwan	Pilot series (S-P 3D E-ALND)	11	No	12	39 (28–49)	10 (8–11)	3	0%	0%	18.1% (temporary)	0%	0%	0% at 7 m	7 months
Wu Z et al. [32]	China	Comparative (RALND vs. E-ALND)	27	No	Yes	43.4 ± 12.4	17.9 ± 6.7	3.3 ± 2.4	0%	0%	NR	NR	NR	NR	3 months
Li Z et al. [20]	China	Retrospective comparative (MIAD vs. CAD)	71	No	Yes	47.5 ± 9.0	17.8 ± 7.9	5.6 ± 4.2	NR	0%	NR	Lower vs. CAD at 1 m and 3 m	0%	0% at 16 m	16 months

**Table 3:** Critical appraisal of the current evidence on endoscopic axillary lymph node dissection.

Evidence Domain	Main Evidence	Level/Strength of Evidence	Main Limitations	Current Interpretation
Technical feasibility	Multiple prospective and retrospective series	Moderate	Predominantly single-center studies; learning-curve effects	Well established
Lymph node retrieval	Comparative studies and meta-analysis	Moderate	Heterogeneity in techniques and study design	Appears comparable to conventional ALND
Postoperative morbidity	Comparative studies and meta-analysis	Moderate	Heterogeneous definitions and assessment of complications; predominantly observational evidence	Suggests a potential benefit
Functional recovery	Small comparative and observational studies	Low–moderate	Small samples; heterogeneous assessment of pain and shoulder function	Potential benefit
Cosmetic outcomes	Small comparative studies and case series	Low–moderate	Subjective outcomes; limited standardized patient-reported measures	Probably favorable
Oncological safety	Limited randomized evidence and observational follow-up	Low	Few randomized studies; selected populations; heterogeneous and frequently short follow-up; insufficient mature long-term oncological data	Insufficient to establish oncological equivalence
Robotic ALND	Small retrospective comparative studies and case series	Low	Small sample sizes; short follow-up; limited availability; costs; lack of cost-effectiveness data	Preliminary
ICG-guided EALND	Small retrospective and technical studies	Very low	Lack of prospective comparative evidence; short follow-up	Experimental/emerging

## Data Availability

No new data were created or analyzed in this study. Data sharing is not applicable to this study.
